# A case report of infantile cystic nephroblastoma

**DOI:** 10.1186/s13000-018-0761-5

**Published:** 2018-10-27

**Authors:** Nozomu Kurose, Michiho Takenaka, Manabu Yamashita, Chie Shimaguchi, Mariko Nakano, Bryant Britni, Xin Guo, Chizuru Futatsuya, Akihiro Shioya, Sohsuke Yamada

**Affiliations:** 1 Department of Pathology and Laboratory Medicine, Kanazawa Medical University, 1-1 Daigaku, Uchinada, Ishikawa 920-0293 Japan; 2 Department of Pathology, Kanazawa Medical University, Ishikawa, Japan; 3 Department of Pathology and Laboratory Medicine, University of Vermont College of Medicine, Burlington, VT USA

**Keywords:** Cyst, Nephroblastoma (NB), Infant, Peritoneal fluid, Cytology

## Abstract

**Background:**

Nephroblastoma (NB) is a malignant embryonal neoplasm derived from nephrogenic blastemal cells. NB usually forms a solid mass, but in extremely rare cases, it may show cystic changes.

**Case presentation:**

A six-month-old girl with persistent high fevers was found to have pyuria and bacteriuria. Ultrasonography revealed multilocular cysts in the right kidney. Right nephrectomy was performed with cyst wall rupture during surgery. An intraoperative rapid diagnosis, based on peritoneal fluid cytology, confirmed three components of blastemal, stromal, and epithelial cells. The blastemal cells were dyshesive, with scant to no cytoplasm and were the predominant cell type. The spindle-shaped stromal cells were arranged in fascicles. The epithelial cells demonstrated tubular structures. Macroscopically, the resected cystic tumor measured 80 mm in maximum diameter with a prominently thin cyst wall, but solid areas were also apparent. Histologically, the tumor was diagnosed as cystic NB (blastemal-predominant) displaying a triphasic pattern. Hyperchromatic nuclei and apoptotic bodies were found. The clinical stage classification of Japan Wilms Tumor Study group was 3. The patient was treated with chemotherapy and radiotherapy. Tumor recurrence and metastasis have not been observed in the 8 months since surgery.

**Conclusion:**

This is an extremely rare case of infantile cystic NB. We diagnosed the NB cells that appeared in the peritoneal fluid by intraoperative rapid cytology. Cytological examination proved to be a very useful technique for determining the clinical stage of NB. Additionally, we propose that massive tumor degeneration and necrosis be considered as a pathogenic mechanism of cyst formation in NB.

## Background

Wilms tumor, also known as nephroblastoma (NB) is a malignant embryonal neoplasm derived from nephrogenic blastemal cells. NB is the most common malignant renal tumor in children and 98% of cases occur under the age of 10. The mean age at diagnosis is 37 months and 43 months among males and females, respectively [1]. However, adult-onset cases have also been reported [2]. Grossly, NB usually forms a solitary and rounded-solid mass. Histologically, the three components of blastemal, epithelial, and stromal cells are mixed in various proportions.

Renal cystic tumors in children can be benign, such as cystic nephroma (multilocular cyst), and malignant homologous tumors, such as cystic partially differentiated NB (CPDNB) [3]. Furthermore, very few case of cystic NBs in infants and adults have been reported [4–6].

We herein report an extremely rare case of cystic NB in an infant and discuss its pathogenic mechanism. In addition, we also describe the cytological findings of NB that appeared in peritoneal fluid.

## Case presentation

A previously healthy six-month-old girl who was born full-term following an uncomplicated pregnancy presented with persistent high fever and was found to have pyuria and bacteriuria. Prior to this, she had no significant medical history. No obvious gross malformations were observed on physical examination nor was any pertinent family history noted. Ultrasonography revealed multilocular cysts in the right kidney; of note, no abdominal abnormalities had been observed at her four-month medical examination.

**Fig. 1:**
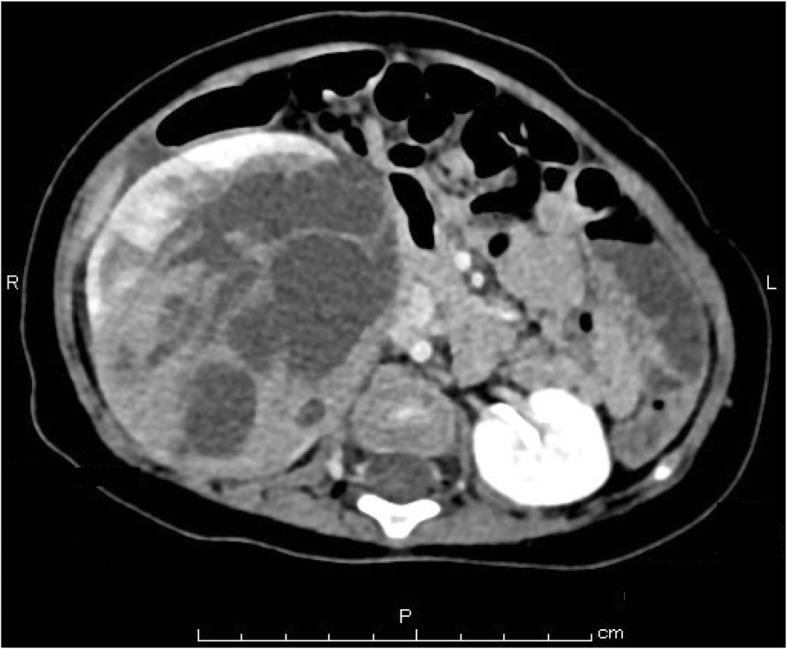
Enhanced abdominal CT. Multilocular cystic mass located in the right kidney. The septal wall of the tumor was thickened. Multiple solid areas were identified within the tumor

**Fig. 2:**
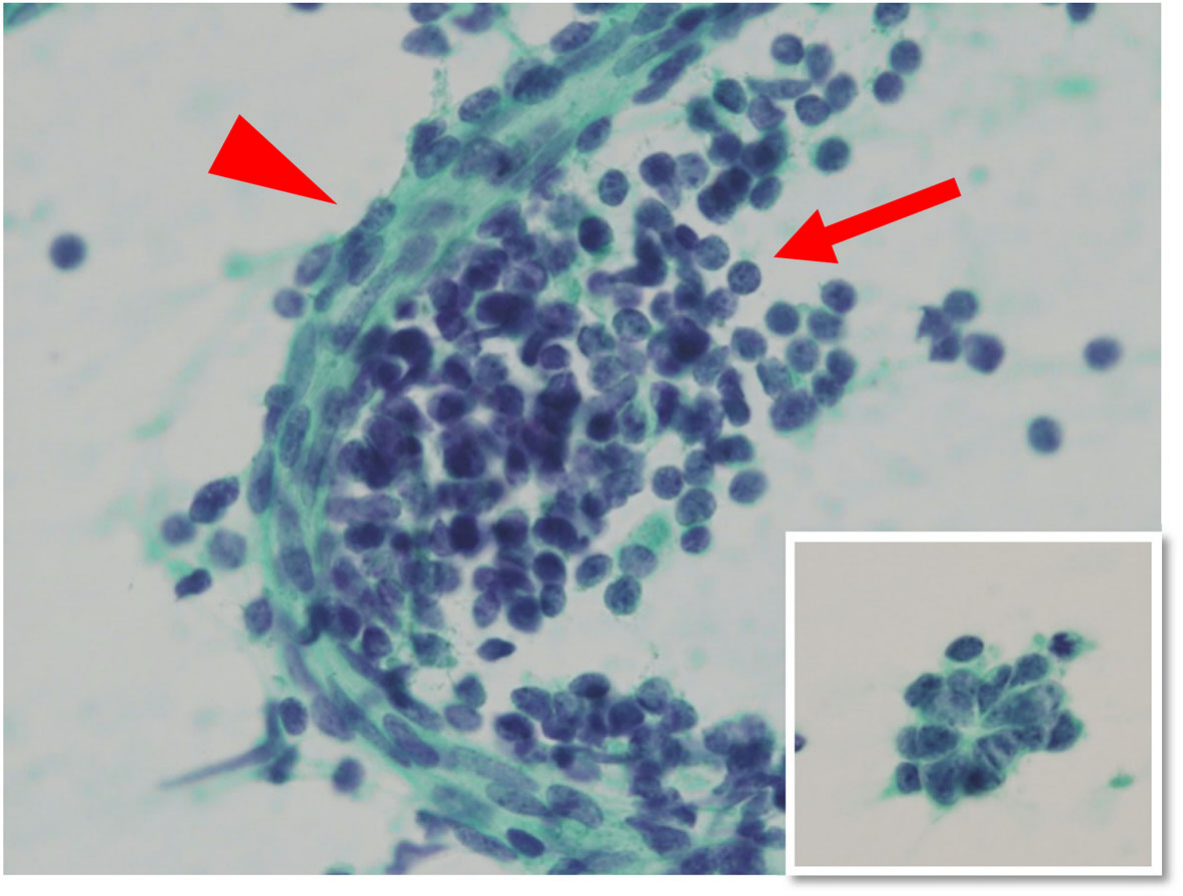
Cytology of the peritoneal fluid. Three components of blastemal, stromal and epithelial cells were seen. The blastemal cells showed decreased cell adhesion (arrow), the stromal cells showed a fibroblast-like configuration (arrow head), and the epithelial cells showed a tubular structure (inset) (Papanicolaou staining, × 400)

Abdominal contrast computed tomography (CT) revealed a multilocular cystic mass accompanied by septal wall formation pressing on the normal kidney parenchyma (Fig. 1). The tumor had not directly infiltrated the renal pelvis and there was no coexistence of hydronephrosis. Renal dysplasia was ruled out due to the presence of adjacent normal kidney parenchyma. Because the cystic septa was thickened, cystic NB was deemed the most likely entity, preoperatively. One month later, right nephrectomy was performed. Unfortunately, the cyst wall ruptured during surgery. An intraoperative cytological evaluation by rapid Papanicolaou staining of peritoneal fluid confirmed three components of blastemal, stromal, and epithelial cells. The findings were interpreted on-site as NB (Fig. 2).

**Fig. 3:**
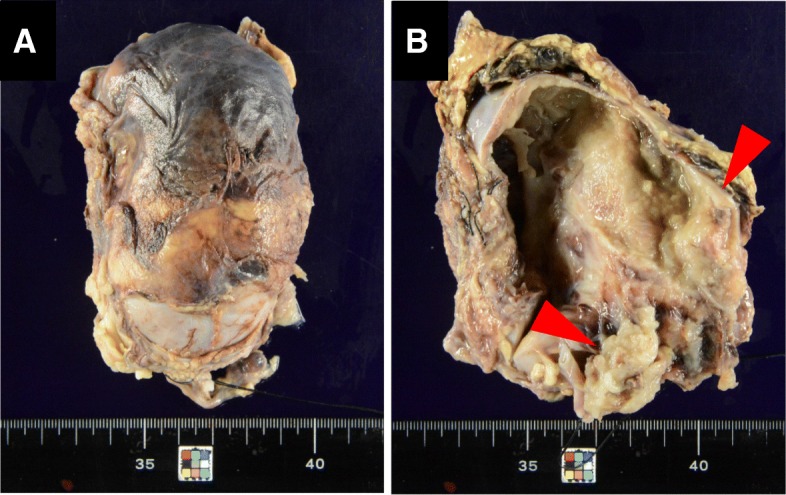
Gross findings of the resected right kidney. **a** The tumor was localized from the upper pole to the middle portion. **b** The cystic septum within the tumor had disappeared. Some solid areas were seen (arrow head)

Cytologically, the blastemal cells were dyshesive, exhibiting naked nuclei with scant to absent cytoplasm and were the most predominant cell type. The spindle-shaped stromal cells were arranged in fascicles, showing a fibroblast-like configuration. And epithelial cells formed tubular structures. Macroscopically, the resected cystic tumor measured 80 mm in maximum diameter involving the upper pole to middle portion of the right kidney (Fig. 3a) and the cystic wall was predominantly thin. A portion of the cystic wall was ruptured by the surgical procedure and was associated with hemorrhage in the surrounding tissues. On the cut surface of the tumor, the cystic septum within the tumor had disappeared (Fig. 3b). Instead, hemorrhage and muddy, degenerative necrotic tumor tissue was found within the cystic space. Solid areas were observed within the cyst walls.

**Fig. 4:**
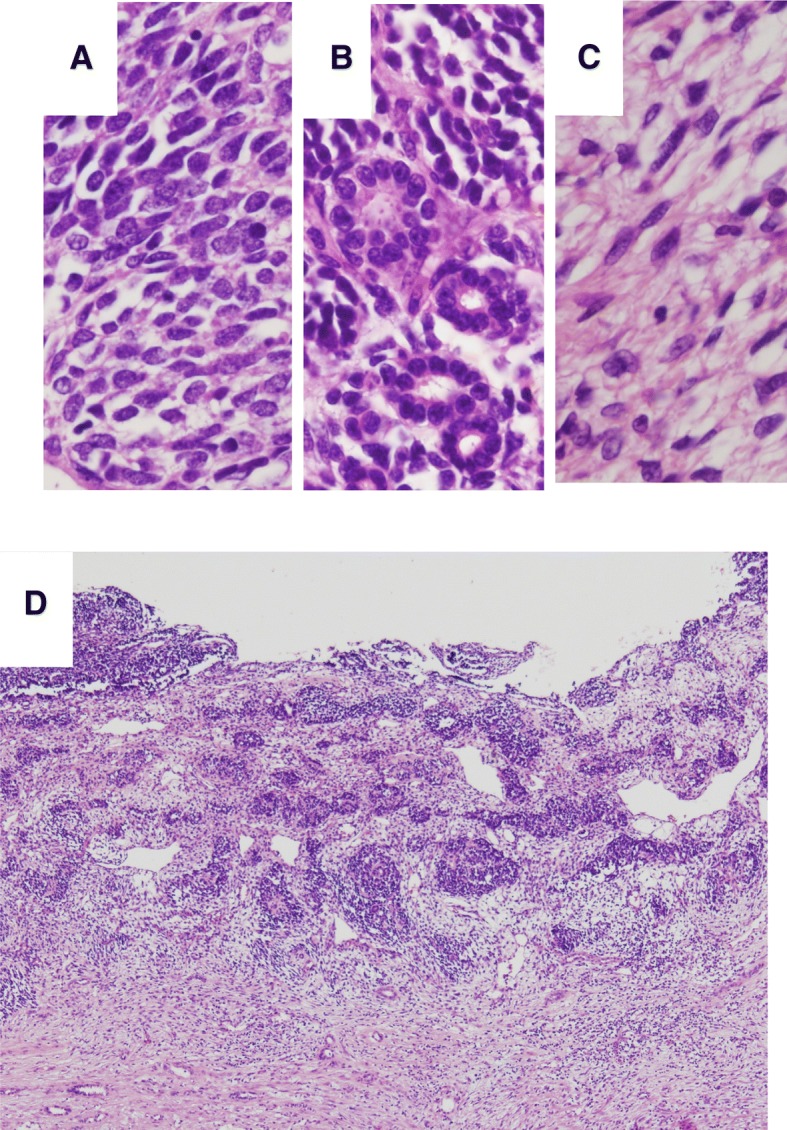
Histological findings of the tumor. **a** Blastemal cells (H&E staining, × 400). **b** Epithelial cells (H&E staining, × 400). **c** Stromal cells (H&E staining, × 400). **c** Low-powered view of cystic wall of the tumor (H&E staining, × 40)

Histologically, the tumor was diagnosed as cystic NB (blastemal-predominant) displaying a triphasic pattern. The blastemal cells showed a solid growth pattern, whereas the epithelial cells showed tubular structures (Fig. 4a, b). The spindle-shaped stromal cells resembled embryonic mesenchyme, displaying differentiation into smooth muscles and fibroblasts (Fig. 4c). The cystic wall was lined by blastemal cells (Fig. 4d). Some solid areas were present within the cyst wall. There were no anaplastic cells or atypical mitoses. Degenerated hyperchromatic nuclei and apoptotic bodies were found in some areas. Immunohistochemically, the blastemal cells and epithelial cells were positive for WT1 (WT49, Leica, diluted 1:1), but the stromal cells were negative. The Ki-67 (MIB-1, BioGenex, diluted 1:30) labeling index of the tumor was as high as 90%, while the degenerated necrotic tumor cells showed decreased staining properties. Nephrogenic rest, a precursor lesion of NB, was not identified in the remaining renal parenchyma. The clinical stage classification of the Japanese Wilms Tumor Study (JWiTS) group was 3. The patient was treated with chemotherapy and radiotherapy and tumor recurrence and metastasis have not been observed in the 8 months since surgery.

## Discussion

This case report describes an extremely rare case of cystic NB that developed in an infant with no family history or malformation. Furthermore, we describe the cytological findings of the tumor observed intraoperatively involving the peritoneal fluid.

The differential diagnosis of cystic NB includes CPDNB. CPDNB also exhibits multiloculated cysts, as in this case, however these lesions should not have apparent solid or nodular mass formation. CPDNB contains mature or immature nephroblastic tissue, but conforms to the septum between cysts. In contrast, cystic NB has solid NB components expanding the cyst wall, which were easily identified in this case.

To our knowledge, only three cases of NB with cyst formation have been reported in the English literature [4–6]. However, there were no case reports of ruptured cystic NB. One case was an infantile case (9.5 months old) and the other 2 cases were in adults (36 and 30 years old). The mechanism underlying the cyst formation is not well understood. However, the following two possible mechanisms have been proposed: 1) formation due to tumor degeneration and necrosis and 2) formation due to the tumor infiltrating into the renal pelvis, leading to urine inflow inside the tumor. In the present case, there was no extensive coagulative necrosis or thrombus formation, nor any apparent perforation between the tumor and the renal pelvis. Although there have been no case reports of spontaneous regression of NB, nephrogenic rest, which is a precursor lesion of NB, exhibits spontaneous regression and eventual scarring as obsolescent rest [7, 8]. In our case, there was no nephrogenic rest or scar tissue in the remaining renal parenchyma. Therefore, it was thought that cyst formation may have been caused by massive tumor degeneration and necrosis of unknown etiology rather than spontaneous regression, given the presence of degenerated tumor cells and apoptotic bodies.

NB may be the cause of abdominal pain, anemia, and shock accompanying tumor rupture. According to the staging system of the JWiTS and the National Wilms Tumor Study (NWTS), diffuse peritoneal contamination associated with spillage of NB cells before and during surgery is classified as stage 3 [9–11]. The NWTS has also reported that spillage of NB cells does not constitute a risk factor after three-drug chemotherapy and whole-abdomen radiotherapy [12]. In addition, the Children’s Oncology Group reported that the incidence of intraoperative tumor spillage increased to 11.9%, and the odds ratio reached 2.183 in patients with a maximum tumor diameter of ≥12 cm compared to those with < 12 cm [13]. In the present case, the maximum tumor diameter was 8 cm, but there was intraoperative tumor rupture associated with tumor cells in the peritoneal fluid. We propose that cystic changes in NB may be a risk factor for tumor rupture. After surgery, the patient received chemotherapy and radiation therapy according to the protocol of the NWTS. Because of the patient’s favorable histologic features (lack of anaplasia) and excellent response to therapy, this case is expected to have a good prognosis.

## Conclusions

We present an extremely rare case of infantile cystic NB. The diagnosis was able to be made on-site by intraoperative rapid cytology of peritoneal fluid. This technique of cytological examination proved to be very useful for determining the clinical stage of NB. Additionally, massive tumor degeneration and necrosis should be considered as a possible mechanism of cyst formation in NB.

## Abbreviations

CPDNB: Cystic partially differentiated nephroblastoma
CT: Computed tomography
JWiTS: Japanese Wilms Tumor Study
NB: Nephroblastoma
NWTS: National Wilms Tumor Study
